# Parliamentary candidates and their campaign messages at the 2019 General Election

**DOI:** 10.1177/02633957231186384

**Published:** 2023-07-27

**Authors:** Siim Trumm, Caitlin Milazzo, Alan Duggan

**Affiliations:** University of Nottingham, UK; University of Nottingham, UK; University of Stirling, UK

**Keywords:** Britain, campaigning, candidates, communication, elections, leaflets

## Abstract

The 2019 General Election brought about a significant change in the parliamentary balance of power. There has already been much attention devoted to how parties and their leaders campaigned in the run up to the polling day. Using original leaflet data from the OpenElections project, this study extends the focus to individual candidates by exploring the nature of local campaign communications. We find that candidates make little effort to promote their personal traits, with personalisation of leaflets remaining largely limited to offering visual cues in the shape of candidate photos. We also find that while negative campaign messages are common, they tend to relate to an opposing party more generally. Similarly, we find that highlighting the tactical situation in the constituency remains a rare practice. Our findings suggest that there is still considerable room for candidates to tailor their campaign materials to their personal attributes and the local electoral context.

## Introduction

The 2019 General Election altered the political landscape of the United Kingdom. The Conservative Party won a landslide majority with the highest percentage of the popular vote of any party since 1979, while the Scottish National Party further strengthened its position in Scotland. By contrast, Jeremy Corbyn decided to step aside as the leader of the Labour Party in the aftermath of the party’s poor election result, and the leader of Liberal Democrats, Jo Swinson, lost her seat of East Dunbartonshire, despite featuring heavily in the party’s national campaign.

There were a number of memorable aspects of the 2019 campaign period: The Conservative Party stood out with its prominent message of ‘Get Brexit Done’ – a strategy that was aided by the Brexit Party’s decision not to field candidates in Conservative-held seats (Ford et al., 2021). And while the Labour Party put forward a wide ranging policy offer, the Liberal Democrats placed themselves in clear opposition to the Conservative Party, with their ‘Stop Brexit’ campaign (Allen and Bara, 2021). Given the perceived importance of the ‘Brexit Election’ in finally realising the United Kingdom’s departure from the European Union, it is unsurprising that there is already a growing body of literature focusing on campaigns preceding polling day. These studies look at parties’ communication strategies (Cooper and Cooper, 2020), the role of party leaders (Evans et al., 2021), coordinated behaviours on social media (Nizzoli et al., 2021), the benefits of local campaigning (Núñez, 2021) and electoral pacts (Mellon, 2021), the use of visual cues in online campaign material (Famulari, 2021), ‘strategic lying’ as a campaign strategy (Gaber and Fisher, 2022), and even the role of masculinity in party leaders’ campaign imagery (Smith, 2021). While the insights we gain from these studies are invaluable in helping us to better understand the outcome of the election, we note that much of the focus of these studies has remained on the *national* campaigns of the parties and their leaders. Their local campaigns and local messaging have been subject to much less scrutiny, even though, ultimately, it is winning local races that determines balance of power in the House of Commons.

Our study uses original leaflet data from the OpenElections project to shed light on the messages that parliamentary candidates put forward in their constituency. Specifically, we explore the extent to which the leaflets that voters received in the run-up to the 2019 election mention individual candidates and, of those that did so, how many feature candidate photos, highlight their personal traits, mention various policy areas, engage in negative campaigning, and discuss the tactical situation in the constituency. In doing so, we not only provide novel insights into the kinds of campaign messages candidates from different parties use, but also contribute to broader debates on campaign personalisation, tactical voting, issue emphasis, and negative campaigning in British general elections. Our evidence suggests that leaflets from parliamentary candidates make up the majority of the campaign literature that voters receive. That said, while almost all candidate leaflets feature at least one photo of the candidate, only a minority of candidates engage in further personalisation by mentioning their links to the constituency or personal traits, such as their educational and employment background. In addition, we find limited evidence to support the perception that either negative campaigning or encouraging tactical voting is widespread, at least in candidates’ campaign communications. While most leaflets do include an attack of an opposing party, few personally mention a leader of an opposing party or an opposing candidate. With regard to encouraging tactical voting, only one in four candidate leaflets use messages of this nature. These findings shed further light on the kind of campaign messages voters are exposed to prior to polling day.

The article is organised as follows. In the next section, we survey the existing literature on campaigning and voting at the 2019 General Election. We then explore themes and literature related to election leaflets, describe the data, present the findings, and conclude with a discussion of their broader implications.

## The 2019 General Election campaign and its consequences

The conventional understanding is that, despite the growing importance of affective polarisation in influencing the political choices of some voters (e.g. Hobolt et al., 2021; Kalla and Broockman, 2018; Mason, 2018), what happens during the campaign period can play an important role in determining how voters cast their ballot. Campaigns matter not only in both established and newer democracies, but also in contests carried out under different electoral rules (e.g. Fisher et al., 2019; Jacobson, 2015; Trumm, 2018). The importance of campaigns is accentuated by a rise in floating voters and the growing relevance of short-term factors, such as evaluations of the economy and/or political leaders, in shaping vote choice (e.g. Dassonneville, 2016; Mellon et al., 2018).

It is, therefore, unsurprising that there is already a notable body of literature focusing on aspects of the 2019 General Election campaign. Many studies explore the effects of the campaign on voters. For example, these studies focus on how Boris Johnson’s messaging helped the Conservative Party appeal to voters in the ‘red wall’ seats (Cooper and Cooper, 2020), why the strongly Leave-voting constituency of Dagenham and Rainham as a key target for the Conservative Party did not turn blue (Cruddas, 2020), the importance of strategic issue positioning on Brexit and the appeal of Boris Johnson in helping the Conservative Party to win the radical-right vote (Evans et al., 2021), the scale of tactical voting (Mellon, 2021), and the role of parties’ messaging on Brexit in shaping vote choice (Prosser, 2021). By contrast, other studies put more emphasis on the campaign choices made by parties. They look at ‘strategic lying’ as a campaign strategy (Gaber and Fisher, 2022), the importance of local campaigning (Núñez, 2021) and electoral pacts (Mellon, 2021), campaigns on various social media channels (Famulari, 2021; Power and Mason, 2021), and the impact of digital campaigning on journalistic efforts to inform voters (Dommett, 2021), while the excellent volume by Tonge et al. (2020) brings together a collection of studies that focus on specific aspects of the campaigns in terms of parties, methods, and countries.

The existing insights are invaluable in helping us to better understand the national campaigns during the 2019 General Election and the extent to which their messages resonated with voters. They have, however, been derived from analyses that focus primarily on the national campaigns of parties and party leaders. While both are undoubtedly important in understanding how electoral competition unfolds, as well as why people vote the way they do, party leaders are not the only political elites who campaign prior to election day. Local parliamentary candidates also play a key part in shaping the kind of cues and messages that voters receive during an election campaign. Therefore, it is important to extend the focus to individual candidates when exploring the nature of general election campaigns.

## Campaign themes in election leaflets

There is a limited amount of research on the content and campaign themes used in election leaflets. This is largely due to the absence of data that can be used for large-scale content analysis. However, there are some notable examples of such analyses in the literature. There has been significant focus on the personalisation of campaign leaflets (Däubler and Ó Muineacháin, 2022; Milazzo and Hammond, 2018; Milazzo and Townsley, 2020) and the use of negativity in campaign leaflets (Duggan and Milazzo, 2023; Milazzo et al., 2021). This article will look at four themes related to the content of election leaflets (1) personalisation, (2) issue emphasis, (3) negativity, and (4) prevalence of tactical voting cues. As such, we offer the first analysis on the prevalence of tactical voting messages in election leaflets and we make a significant contribution to a limited literature on issue emphasis. In the cases of personalisation and negativity, we offer new insights in this literature by exploring disaggregated versions of these variables.

Previous work has linked the personalisation of election leaflets to local and national factors. With respect to the local context, the personalisation of electoral campaigns has been linked to the candidate’s perception of her electoral chances, suggesting that candidates in safer seats and those that expect to win, such as incumbents, are more likely to personalise their leaflets and campaigns (Milazzo and Townsley, 2020; Townsley et al., 2022). In addition to a candidate’s personal circumstances, aspects of the party’s national fortunes may influence the decision to personalise one’s local campaign or not. Däubler and Ó Muineacháin (2022) find that candidates are more likely to personalise their leaflets if the brand of their party is unpopular with the electorate, while Milazzo and Townsley (2020) find that candidates whose party leader is perceived to be unpopular will be more likely to use personal messages in their leaflets. To add to this work, we explore the prevalence of personalisation using more nuanced categories of personalisation than previous work on election leaflets – that is education, family, employment, and locality – which gives us a more detailed understanding of the type of personalisation used in election leaflets.

Previous work on the use negativity of British election leaflets has shown that seat marginality, membership of a governing party, and constituency incumbency have significant effects on the likelihood for candidates to engage in negative messaging (Duggan and Milazzo, 2023). The importance of these factors in predicting negativity is also supported by a significant amount of work from the wider literature on campaign negativity (e.g. Druckman et al., 2009; Elmelund-Præstekær, 2010; Fowler et al., 2016; Nai, 2020). In this article, we disaggregate negativity into three categories: (1) attacks on an opposing party, (2) attacks on an opposing party leader, and (3) attacks on an opposing candidate. These categories vary significantly in how personal the type of attack may be perceived. Previous work has suggested that variability in the perception of how personal/uncivil an attack is can be an important determinant of its effect (Haselmayer, 2019). As such, we explore the prevalence of each of these types of negativities to offer a better understanding of their distribution in British election leaflets.

There has been a limited focus in the literature on issue emphasis or the prevalence of tactical voting in British election leaflets.^1^ As such, our analysis offers novel insights into these types of leaflet content. We expect that issue emphasis will be significantly influenced by the election that is being studied – that is we would expect a significant emphasis on Brexit in 2019. In addition, we would expect to see inter-party differences in terms of issue emphasis. It is likely that the prevalence of issues in leaflets is influenced by issue ownership (Walgrave et al., 2015). In other words, parties will highlight issues on which the public perceive them as being competent and will attempt to steer away from issues on which they are seen as weak. We explore issue emphasis by party and find some notable results to support this assumption.

Finally, we explore the prevalence of tactical vote messaging in the 2019 election. Tactical voting in British general elections has been a topic of interest in the literature on campaigns going back to the 1980s (e.g. Galbraith and Rae, 1989; Johnston and Pattie, 1991). More recent work has shown that voters supporting uncompetitive parties are more likely to vote tactically when they receive clear signals and information on the competitiveness of the race in their constituency (Kiewiet, 2013). As such, it is important to study the prevalence of tactical voting messages in election leaflets so we can understand how candidates tailor their campaign messages. We would expect the frequency of such messages to be influenced by the marginality of the seat. For each of the four campaign themes discussed in this section, we look at the results in aggregate terms, by party, and by candidate competitiveness. This allows us to tease out some of the nuance that characterises British election leaflets.

## Data and methods

We use original leaflet data from the OpenElections project (www.openelections.co.uk) to examine the nature of parliamentary candidates’ campaign communications in the run-up to the 2019 General Election. The OpenElections data is well suited for improving our understanding of the campaign messages voters receive locally. First, it is the largest collection of unsolicited British campaign communications – that is election leaflets – in existence. The collection includes leaflets from all major political parties, from all three nations of Great Britain, and comprises leaflets distributed in the immediate run-up to polling day (i.e. during the long and short campaign periods), when voters tend to pay most attention to politics.

Second, the leaflets in the OpenElections dataset are coded on several dimensions, including candidate and party leader mentions, references to various personal traits and issue areas, opponent attacks, and mentions of the tactical situation in the constituency. Taken together, these data not only tell us what parties and candidates talk about, but also who they talk about and how they do so. Finally, during the 2019 General Election, parties and their candidates spent more than £36 million on unsolicited communications, which is more than they spent on any other activity.^2^ On this basis, it is unsurprising that receiving an election leaflet was the most common form of campaign contact reported by respondents of the British Election Study (BES) following the 2019 election. Of those who reported that they had been contacted by a party in the final weeks of the campaign, nearly 90% indicated they had received a leaflet or a letter from at least one party (Fieldhouse et al., 2021).^3^ Given the importance of these communications, studying the messages contained within them has the potential to provide important insights into the nature of the election campaign. Moreover, it is a form of contact that reaches voters at their home and, therefore, it can potentially reach even those voters who are uninterested in politics and do not wish to actively seek out information about parties and candidates.

The full OpenElections dataset contains nearly 9000 leaflets from all major political parties since 2010 and covers 600 out of the 632 constituencies that have been in use during this period. For the current article, we focus on leaflets distributed during the 2019 General Election by parliamentary candidates from the Brexit Party, the Conservative Party, the Green Party, the Labour Party, Liberal Democrats, Plaid Cymru, and the Scottish National Party (SNP). As we are, first and foremost, interested in the campaign messages put forward by individual candidates, we identify whether a leaflet mentions a party’s candidate by name. The cost of any unsolicited materials that do so is counted against the candidate’s election spending. Therefore, by treating leaflets where the candidate is mentioned by name, we can obtain a conservative estimate of ‘candidate leaflets’.

The 2019 sample includes a total of 1223 leaflets, with 924 being candidate leaflets.^4^ Table 1 provides a breakdown of all 2019 leaflets by party, the percentage of each party’s leaflets that mention its candidate by name and the breakdown of candidate leaflets by party. Approximately three in four (75.6%) of all the 2019 leaflets include the name of the party’s candidate. The notable outlier here is the Brexit Party, which was the only party where campaign communications were more likely to *not* mention their local candidate than to do so (63.3% vs 36.7%). This is unsurprising, given that the party had only registered to run candidates earlier in 2019 and its key electoral asset was arguably its then-leader Nigel Farage. The majority of leaflets distributed by all the other parties mention their local candidates by name, ranging from 64.3% for Liberal Democrats to as high as 95.4% for the Green Party.

**Table 1.** Distribution of all leaflets and candidate leaflets by party.

	All leaflets			Candidate leaflets	
	Count	%	Candidate mentions (%)	Count	%
Brexit	60	4.9	36.7	22	2.4
Conservative	336	27.5	80.0	268	29.0
Green	87	7.1	95.4	83	9.0
Labour	328	26.8	85.1	279	30.2
Lib Dem	373	30.5	64.3	240	26.0
National	39	3.2	82.1	32	3.5
All	1,223		75.6	924	
Constituencies				240	38.0
Mean N per constituency				3.9	
Range				[1,19]	

The reliance on leaflet data from the OpenElections project offers unique research opportunities, but also calls for caution. The dataset is a sample of convenience and, given that parties and individual candidates are not required to report how many leaflets they distribute, we cannot determine how representative the sample is of the total population of leaflets distributed. That said, there is no reason to believe that our sample is biased. There are no incentives or institutions encouraging people to upload leaflets to the OpenElections repository, the project has no partisan affiliations, and it has received no funding from non-academic sources. Moreover, when we compare the OpenElections leaflet data with contact rates reported in the BES, we find, for all parties, a positive and statistically significant correlation (0.20, *p* < .01) between the number of leaflets we have for a candidate and the percentage of BES respondents in the same constituency who reported receiving a leaflet from the same party in the run up to the polling day.

### Campaign messaging

We focus on four different aspects of campaign messaging in our empirical analysis. First, we capture campaign personalisation through two indicators. *Candidate photo* distinguishes between candidate leaflets that feature at least one image of the candidate (coded 1) and those with no images of the candidate (coded 0). In addition, we examine whether candidate leaflets mention candidates’ *education, employment, family*, and *locality*. These four variables are coded 1 if the personal trait is mentioned in a leaflet, and 0 if not. Taken together, these variables provide a valuable overview of the extent to which leaflets are personalised and, if so, what kind of personalised features are more common than others.

Second, we capture whether candidate leaflets discuss certain policy areas. This provides us with an insight into what different candidates focus on. Candidate leaflets are coded on whether they talk about issues relating to *the economy, education, environment, Europe, health, immigration*, and *welfare*, or not, with the variables coded 1 if the respective policy area is mentioned in the leaflet, and 0 otherwise.

Third, we explore the extent to which negative campaign messages are used in leaflets. *Party attack* distinguishes between candidate leaflets that include at least one negative mention of an opposing party (coded 1) and those that do not (coded 0). For further nuance, we also capture *party leader attack*, describing whether the leaflet includes any negative mentions of at least one opposing party leader (coded 1) or not (coded 0), and *candidate attack*, which distinguishes between leaflets with at least one negative mention of an opposing candidate (coded 1) and those with none (coded 0). Taken together, the three indicators provide insight into the overall extent to which negative campaigning is used, as well as more personalised attacks.

The final aspect of campaign messaging that we focus on is the extent to which candidate leaflets draw voters’ attention to the electoral context in their constituency. We do so through *tactical situation*. It captures whether the leaflet highlights the tactical situation in the constituency (coded 1) – for example, ‘Labour can’t win here’ – or not (coded 0).

### Partisanship

Leaflets are also coded for *party*. We distinguish between leaflets from candidates running for the Brexit Party, the Conservative Party, the Green Party, the Labour Party, Liberal Democrats, and national parties (Plaid Cymru and the SNP). This allows us to tease out party-specific variation in candidates’ campaign messaging.

## Findings

We now turn to the empirical analysis to examine the extent to which different campaign messages appear in leaflets, and whether there is variation in how the candidates of different parties attempt to persuade voters. First, we explore further the nature of personalisation in election leaflets, which candidates could achieve via visual cues (i.e. personal photos) or by mentioning their personal traits. Table 2 presents the percentage of candidate leaflets that feature a candidate photo, as well as the percentage that mentions a range of personal attributes.

**Table 2.** Percentage of candidate leaflets with personalised features.

	All	Brexit	Conservative	Green	Labour	Lib Dem	National
Candidate photo	91.7	26.7	71.7	96.6	84.5	60.6	84.6
Candidate traits							
Education	6.8	18.2	5.6	2.4	7.2	8.8	3.1
Employment	34.6	50.0	25.4	43.4	30.1	45.0	40.6
Family	14.1	13.6	11.6	10.8	17.2	14.6	12.5
Locality	30.6	40.9	17.5	32.5	37.3	37.9	15.6

The story that emerges is interesting. On one hand, it is very common for candidate leaflets to include at least one candidate photo. The Brexit Party stands out as an exception – only 26.7% of their leaflets include a candidate picture, while a majority of leaflets from the candidates of other parties do so. Liberal Democrats opted for a highly leader-centred campaign, focusing heavily on their then-leader Jo Swinson and her pitch to become the next Prime Minister, which is reflected in the fact that only 60.6% of their candidate leaflets feature a candidate photo. The figures for the other parties all exceed 70%, and in the case of the Green Party, the inclusion of a candidate photo is nearly universal (96.6%).

While it is common for candidate leaflets to feature a personal photo, it is rare for candidate leaflets to highlight personal traits. Only a minority of candidate leaflets mention their educational background (6.8%), employment (34.6%), family (14.1%), and even their ties to the constituency (30.6%) are rarely discussed. There is, however, party-level variation, with Brexit Party candidates generally being more likely to draw attention to these features and Conservative Party candidates being the least likely to do so. That so few candidate leaflets talk about a candidate’s personal background is counterintuitive. For example, being a local candidate often acts as a signal of the ability of a candidate to understand local concerns and tends to be electorally rewarded by voters (e.g. Campbell and Cowley, 2014; Jankowski, 2016). Moreover, Cowley et al. (2022) find that MPs are increasingly likely to represent seats in the region where they were born – by 2019, 52% of all MPs, and 64% of new-elected MPs, were local. And while there were clear differences between parties, their findings suggest that there is a general move towards nominating more local candidates. It is, therefore, surprising that candidates are not emphasising their local ties more prominently. Fewer than one in three leaflets do so (30.6%), and those from candidates of the Conservative Party and national parties stand out as particular unlikely to do so at 17.5% and 15.6%, respectively. At the other extreme are leaflets from the Brexit Party candidates at 40.9%, followed closely by leaflets from Liberal Democrat and Labour candidates at 37.9% and 37.3%, respectively. In sum, campaign personalisation remains, to a large extent, limited to referencing the candidate’s name and adding a personal photo, while detailed information about personal traits remains a rare feature in candidate leaflets.

We turn our attention now to the issue areas that candidates emphasised in their leaflets. Table 3 presents the percentage of candidate leaflets discussing different topics, both overall and by party. The three issue areas most likely to be mentioned in leaflets were health (74.1%), Europe (71%), and the economy (70.8%). This is, of course, entirely unsurprising. Health and the economy are issues that are consistently deemed to be among the most salient issues to voters (Fieldhouse et al., 2020). That nearly three-quarters of leaflets (71%) covered mentioned Europe highlights the prominence of questions around a potential second referendum, impact of Brexit on the devolved nations, the ability to ‘get Brexit done’, or to stop it entirely. Indeed, the Lord Ashcroft Polls conducted on the election day revealed Brexit as the third most important factor in determining vote choice (Lord Ashcroft Polls, 2019), demonstrating a close alignment between what parties and their candidates talked about and what voters took into account when casting their ballot. Finally, more than two-thirds of the candidate leaflets (68.3%) also featured a discussion about environment, highlighting the growing saliency of the topic, and 62.7% of the leaflets discussed matters pertaining to education.

**Table 3.** Percentage of candidate leaflets mentioning different topics.

	Economy	Education	Environment	Brexit/Europe	Health	Immigration	Welfare
Brexit	81.8	27.3	0.0	95.5	81.8	54.6	4.6
Conservative	72.4	74.6	44.8	60.1	82.1	6.0	4.9
Green	62.7	16.9	100.0	74.7	30.1	3.6	10.8
Labour	76.0	67.4	77.8	69.2	82.1	1.4	43.4
Lib Dem	65.4	67.5	81.3	80.4	73.3	0.4	5.0
National	65.6	28.1	50.0	81.3	53.1	0.0	50.0
All	70.8	62.7	68.3	71.0	74.1	3.9	18.6

In terms of the other policy areas, both immigration and welfare featured less frequently in candidate leaflets. Fewer than 1 in 10 (3.9%) discussed aspects of immigration policy, and 1 in 5 (18.6%) mentioned welfare. There is, of course, some party-level variation, with immigration featuring in most of the Brexit Party candidates’ leaflets, and welfare being regularly covered in leaflets disseminated by candidates of the Labour Party and national parties. Thus, it is clear that some policy areas were more central to the local campaigns ahead of the 2019 General Election.

Beyond issues, negativity is seen as an increasingly prominent feature in contemporary British political discourse. Among voters, it is evident that declining trust in politicians and the perception that politicians do not care what their constituents think are worryingly common (Clarke et al., 2018; Fieldhouse et al., 2020), and such voter sentiments have been linked to the effects of campaign negativity (Lau et al., 2007). On the contrary, the electoral ‘success story’ of negative campaigning in Britain goes back for decades (Yoon et al., 2005). Table 4 presents the percentage of candidate leaflets with at least one attack on (1) an opposing party, including its leader or candidate, (2) a leader of an opposing party, and (3) a candidate of an opposing party.

**Table 4.** Percentage of leaflets mentioning an opponent.

	Party	Party leader	Candidate
Brexit	50.0	31.8	4.6
Conservative	65.3	48.9	1.9
Green	13.3	0.0	1.2
Labour	77.8	14.0	3.2
Lib Dem	80.4	55.8	7.5
National	75.0	43.8	0.0
All	68.3	35.2	3.7

The narrative surrounding negative campaigning in the 2019 election leaflets is rather mixed. The evidence does suggest that a majority of candidate leaflets included negative messages, with more than two in three (68.3%) doing so. There is some perhaps unsurprisingly party-specific variation, with the Green Party leaflets being least likely to include negative campaign messages by a considerable margin. As few as 13.3% of leaflets from the Green Party candidates included a negative message, suggesting a general rejection of negative campaigning. However, they remain very much an outlier. A clear majority of leaflets from the candidates of all other parties featured an attack on an opposing party.

The story changes when we focus on attacks of individuals. The percentage of leaflets with a negative mention of an opposing party leader drops to 35.2%. Leaflets from some parties were more likely to include such attacks – that is the Liberal Democrats at 55.8% and the Conservative Party at 48.9%. That said, there are many parties that appear less likely to use these types of messages, and the overall frequency of party leader attacks is significantly lower than the frequency of party attacks. The move away from negativity is even more pronounced when looking at candidate attacks. It is very rare for leaflets to attack an opposing candidate, with only 3.7% of the candidate leaflets in our dataset including such a message. It is most common in Liberal Democrats leaflets, where 7.5% of leaflets include an attack on an opposing candidate. Negative messaging is undoubtedly common in leaflets, but one should be cautious about its prominence. The strategy is largely restricted to targeting opposing parties, with less focus on individual politicians from an opposing party.

Next, we explore how common it is for campaign communications to make a reference to the tactical situation in the constituency. There has been much talk recently about the perceived rise in tactical voting, for example, in the context of the 2022 by-elections in Wakefield and Tiverton and Honiton. The improved ease of tactical voting due to the rise of websites such as www.tacticalvote.co.uk has also increased attention on this campaign tactic. Of course, it is not only voters who may use tactical considerations to guide their electoral behaviour, as evident in the Unite to Remain pact and the Brexit Party’s decision to stand aside in favour of incumbent Conservative candidates at the 2019 General Election.

Table 5 presents the percentage of leaflets that mentioned the tactical situation in the constituency. The evidence shows that such mentions are not particularly common in campaign communications. Overall, around one in four leaflets (26%) highlight the tactical situation in the constituency. These leaflets encourage two types of tactical voting – the first would most commonly be a challenger leaflet that portrays the challenger as best able to defeat an existing incumbent and asks supporters of a third party to lend their votes to them. The second type would generally be an incumbent leaflet that highlights the close nature of the race in the constituency and asks supporters to turn out in order to avoid a defeat by a challenger. Therefore, while a substantial minority of leaflets do use tactical vote messaging as a strategy, there is no evidence here to suggest that it is used widely.

**Table 5.** Percentage of leaflets mentioning tactical situation in constituency.

	Tactical situation
Brexit	27.3
Conservative	15.3
Green	13.3
Labour	25.1
Lib Dem	45.4
National	9.4
All	26.0

However, once again, we do observe variation in the use of tactical messaging by the candidates of different parties. Leaflets disseminated by Liberal Democrats’ candidates were the most likely to reference the tactical situation, with nearly half of them (45.4%) doing so. Interestingly, while the common message across these leaflets was to highlight the Liberal Democrat candidate as best placed to defeat the incumbent, the ‘targets’ were relatively balanced and included seats held by both Conservative and Labour Party incumbents. In addition, just over a quarter of leaflets from the Brexit Party candidates (27.3%) and the Labour Party candidates (25.1%) also did so. In terms of the remaining parties, their leaflets were very unlikely to refer to the tactical situation in the constituency.

Finally, we examine whether candidates’ campaign messages are influenced by the electoral context. It has been shown that constituency marginality and expected likelihood of success can influence electoral campaigns in Britain and beyond (e.g. Evens et al., 2017; Jonston et al., 2012; Trumm and Sudulich, 2019). It is possible that it might also have influenced the kind of campaign messages that different candidates put forward in 2019. For example, it would make sense for candidates in close races to be particularly likely to highlight the tactical situation in the constituency to motivate their own supporters to turn out and encourage the supporters of candidates with no real chance of winning the seat to lend them their vote. Table 6 compares the campaign messages included in leaflets from candidates in competitive races with those put forward by candidates who were not.^5^

**Table 6.** Campaign messaging in different races.

	Competitive (%)	Safe (%)	*p* value
Personalisation			
Candidate photo	85.7	93.3	.00
Candidate trait: education	8.4	6.4	.32
Candidate trait: employment	33.0	35.1	.57
Candidate trait: family	12.8	14.4	.55
Candidate trait: locality	23.2	32.8	.01
Policy areas			
Economy	69.5	71.1	.65
Education	62.6	62.6	.98
Environment	56.2	71.7	.00
Brexit/Europe	59.6	74.2	.00
Health	73.4	74.3	.79
Immigration	3.9	3.9	.97
Welfare	27.6	16.1	.00
Negative campaigning			
Party	72.9	66.9	.11
Party leader	34.5	35.3	.83
Candidate	2.5	4.0	.30
Tactical situation	28.6	25.3	.35
Number of leaflets	203	720	–

The results indicate that the campaign messages included in the leaflets of candidates in competitive and safe races are very similar. Leaflets from candidates in competitive races are slightly more likely to refer to the tactical situation in the constituency and attack an opposing party, but these differences remain marginal and do not reach the conventional level of statistical significance. Similarly, the differences in the likelihood that a leaflet attacks an opposing party leader or candidate remain small. We do, however, find some significant differences when looking at the extent to which leaflets include personalised messages and focus on various policy areas. Candidates in safe races are more likely to use their photo in their campaign communication (93.3% vs 85.7%) and they are also more likely to highlight their local ties (32.8% vs 23.2%). With regard to policy areas, they are more likely to talk about environment (71.7% vs 56.2%) and Brexit/Europe (74.2% vs 59.6%), but less likely to talk about welfare (16.1% vs 27.6%). These findings suggest that there are minor differences in the campaign messaging of candidates in competitive races and those who are not. That said, these differences are confined to a limited number of aspects and, even when present, remain small. Constituency marginality and close races may incentivise greater campaign effort, but this evidence suggests it had limited impact on the content of candidates’ campaign communications in the run-up to the 2019 General Election.

## Discussion: Implications of the findings

The 2019 General Election was highly influential in shaping the political situation in the United Kingdom. It led to the largest Conservative majority since the 1980s, with the party receiving a strong mandate to ‘get Brexit done’, but also leadership contests in the Labour Party and Liberal Democrats. Moreover, the election campaign appeared to further emphasise trends that are increasingly part of the political commentary in Britain, from the perceived personalisation of politics as evident in the prominence of Boris Johnson and Jo Swinson in their parties’ campaign communications to the rise in negative campaigning and encouraging tactical voting as increasingly common election strategies, at least on the national level.

Our study uses leaflet data from the OpenElections project to study the nature of campaign communications that voters received from local candidates. It explores the extent to which leaflets – still the most common form of election communication – are personalised to the candidate, talk about different policy areas, engage in negative campaigning, and refer to the tactical situation in the constituency. We find that most candidates do personalise their leaflets, but only to a degree. Leaflets are highly likely to mention the candidate by name and, if they do so, to also include an image of the candidate. Mentioning personal traits of the candidate like their educational background or local ties, however, remains rare, even in candidate leaflets. In addition, we find that most candidate leaflets include an attack of an opposing party, but it is rare for them to target an opposing party leader or an opposing candidate personally, and that it is still relatively rare for leaflets to highlight the tactical situation in the constituency.

Our analysis of disaggregated measures of personalisation and negativity advances the existing literature by offering a more granular understanding of content variation within candidate leaflets. Moreover, this article adds to a limited literature on issue emphasis in election leaflets and offers the first analysis in this area in over 10 years (Fisher, 2005; Shephard, 2007). As such, our findings offer a novel contemporary understanding of constituency-level variation of issue emphasis. Our analysis highlights the increased prevalence of issues, such as Brexit and climate change, in the 2019 election while also providing evidence of the role of issue ownership in shaping interparty variation in content (e.g., the Labour Party’s comparatively high focus on social welfare). Taken together, these findings shed further light on the kind of campaign messages voters tend to be exposed to in British elections.

There are three broader points arising from this study. First, the findings imply that the personalisation of locally distributed campaign communications remains relatively limited. The personal profiles of candidates do not tend to feature prominently in election leaflets. Voters are likely to find out the name of the candidate and, if so, also see a photo of the candidate, but they are unlikely to learn much about the personal background of the candidate. We know that personal traits such as candidate’s local ties and occupation can have sizable effects in Britain on their electoral appeal (Campbell and Cowley, 2014), but they are still relatively uncommon features in leaflets. It does appear that there is room to extend the kind of personalisation of campaign material that voters receive, and to do so in a manner that could have a positive effect on candidates’ electoral performance.

Second, the findings suggest that local election campaigns in Britain are still not particularly negative. There has been a lot of discussion recently about voters being very disillusioned with politicians and politics more broadly, with negative sentiments both increasingly common and strong. In other words, the relationship between voters and politicians does not appear to be particularly healthy. We know that negative campaigning can contribute to such views as they can create a more negative view of the target, a backlash against the source (Galasso et al., 2021), and increased cynicism more broadly (Mutz, 2015). The relative lack of prominent negative messaging in election leaflets does imply that we need to look elsewhere for the main drivers of political distrust and disillusionment in Britain.

Third, it appears that most parties have not yet appreciated the potential importance of tactical voting. There is a lot of talk about establishing a ‘progressive alliance’ and electoral pacts in general, as well as many voters being increasingly interested in the electoral outlook of their constituency and being willing to ‘lend their vote’. However, our analysis suggests that it is still rare for most parties and their candidates to encourage this type of behaviour. Our findings on tactical voting are novel within the literature. In addition, the constituency level nature of our data allows us to get a much greater sense of the actual prevalence of this strategy across the country and to offer new analytical insights into the phenomenon of tactical voting. Accordingly, it does appear that the rise in floating voters and tactical voting is primarily a bottom-up, rather than top-down, process. From parties’ and candidates’ perspective, this presents an opportunity that political elites may wish to utilise more regularly in future elections through additional references to the tactical situation in the constituency.

Given the lack of work on the content of election leaflets generally, there are several fruitful avenues for future research in this area. First, there is potential to expand our understanding of issue emphasis in election communications. Using a text analysis approach to estimate issue emphasis in election leaflets (Klüver and Bäck, 2019), it would be possible to provide a more granular understanding of *how much* of a leaflet is devoted to particular issues. Such an analysis would offer valuable insights into how responsive candidates are to issues that are considered most important by their constituents. It would also provide a significant contribution to our understanding of how candidates craft their campaign messages around issues and how their priorities reflect the electorate. Second, there is scope to improve our understanding of negativity in election leaflets. Previous work has emphasised the importance of degrees of negativity in determining voter perception and reaction (Haselmayer, 2019; Mattes and Redlawsk, 2014). A text analysis approach in this area would allow for a greater understanding of *how much* of a leaflet is given over to negative messaging and *how* negative the language is. Third, there are many unexplored avenues of research in estimating differences in leaflet content based on candidate characteristics. One such avenue would be to investigate differences in content based on candidate’s gender. Such analyses would offer us a greater understanding of content variation than currently exists in this literature. This would also link the literature on election leaflets to similar analyses that have been carried in other mediums, for example work on gender differences in German leader television debates (Boussalis et al., 2021).

In sum, these findings extend our understanding of the kind of cues and messages voters in Britain were exposed to in the run-up to the 2019 General Election. They not only provide novel insight into the campaign communications put forward by individual candidates, but also speak of some of the broader themes that are increasingly salient in the contemporary British political discourse, such as the personalisation of politics and negative campaigning.

## Supplemental Material

sj-docx-1-pol-10.1177_02633957231186384 – Supplemental material for Parliamentary candidates and their campaign messages at the 2019 General Election Supplemental material, sj-docx-1-pol-10.1177_02633957231186384 for Parliamentary candidates and their campaign messages at the 2019 General Election by Siim Trumm, Caitlin Milazzo and Alan Duggan in Politics

## Notes

1. Notable exceptions with regard to issue emphasis include Shephard (2007) and Fisher (2005).

2. Figures are taken from the Electoral Commissions website (www.electoralcommission.org.uk) (accessed 4 August 2022).

3. The percentage of respondents who reported receiving a leaflet or letter far exceeds the percentage of respondents who reported contact via email (22%), social media (13%), telephone (7%), or canvassing (27%).

4. Please see the Supplementary Appendix for further information about data collection and the sample.

5. We define competitive candidates as those who run in a constituency where the margin of victory at the 2017 General Election was below 10% and whose party was either the winner or the runner-up in 2017 in their constituency.
