# Supplementary material for: Parliamentary candidates and their campaign messages at the 2019 General Election
Source: Politics (Oxf). 2023 Jul 27;45(2):314–30. doi: 10.1177/02633957231186384 (PMC11934510; doi:10.1177/02633957231186384)
Supplement: sj-docx-1-pol-10.1177_02633957231186384 – Supplemental material for Parliamentary candidates and their campaign messages at the 2019 General Election [file sj-docx-1-pol-10.1177_02633957231186384.docx]

**Supplementary Appendix for**

“Parliamentary candidates and their campaign messages at the 2019 General Election”

# **Data gathering and coding**

Data are taken from the OpenElections project (www.openelections.co.uk) – the largest dataset of British campaign communications in existence. The full dataset contains around 9,000 leaflets from all major political parties competing during the last four general elections, and covers 600 out of the 632 constituencies that have been in use since 2010. Leaflets used in the OpenElections project are crowd-sourced, uploaded either to the record-keeping website Electionleaflets.org (www.electionleaflets.org) or directly to the OpenElections website. The former is run by a non-profit, volunteer organisation that aims to create an online repository of leaflets. The latter is an academic project, funded by the Biotechnology and Biological Sciences Research Council, and aimed at analysing the content of leaflets. Both sites are non-partisan.

Leaflets used in the OpenElections database are manually coded on policy dimensions such as the economy, education, Europe, immigration, health, etc. With exception of Europe and immigration, all issue dimensions are coded using the topics and subtopics used by the UK Policy Agendas Project. In addition to policy issues, the dataset identifies whether a leaflet focuses on the traits of the individual candidate, if the leaflet includes a photograph of the candidate, if the leaflet criticises other parties, their leaders or candidates, and if the leaflet mentions the tactical situation in the constituency.

Due to the flexible size and content of electoral leaflets, a single leaflet may include multiple policy statements, mentions of opposing parties, or other forms of potentially interesting content. For example, a leaflet distributed in Broxtowe in the 2019 general election states that the party will:

- “Give patients the care they need through increase NHS funding, more doctors and nurses and an end to privatisation” (health)
- “Invest in our children’s futures with National Education Service that is free for everyone” (education)
- “Kickstart a Green Industrial Revolution to tackle the climate emergency…” (environment) “…and create high skilled, high wage jobs” (economy)
- The leaflet also makes references to opponents (“Stop Boris Johnson’s disastrous Brexit deal and give the people the final say”)

Leaflets uploaded to Electionleaflets.org were manually coded by a single researcher on all dimensions used in this paper, using a pre-defined set of coding instructions. For issue areas, candidate mentions, and reference to a tactical situation, newer leaflets uploaded to the OpenElections website were initially coded via crowdsourcing, using simple, pre-defined instructions. The coding was then checked by a researcher against these instructions. For candidate photos, candidate traits, and disaggregated forms of attacks on an opponent, newer leaflets uploaded to the OpenElections website were coded by a single researcher. This means that a large majority of the leaflets were coded by a single researcher. For a sizeable minority, leaflets went through two phases of coding (i.e., crowdsourced and researcher review) on a subset of variables. For the 2019 election, 1,127 leaflets (91.5%) were coded by a single coder, while 105 (8.5%) were crowd coded on a subset of variables and then reviewed by a researcher. In general, we see reassuring consistency when comparing the dimensions for which we have both types of coding (see Table S1 below).

[TABLE S1 HERE]

# **Evaluating the potential bias associated with self-selected data**

The data cover a wide range of seats in England, Scotland, and Wales, but we acknowledge it is a sample of convenience. To evaluate the potential differences between the population of constituencies and the sample of constituencies used in our analysis, we conduct a series of t-tests to identify any systematic differences. Table S2 compares the mean values for the constituencies that are included in our dataset versus those constituencies where we have no candidate leaflets on multiple dimensions, as well as the associated p-values. A p-value below 0.05 suggests that constituencies for which we have data differ meaningfully from those that are absent from the dataset on that trait.

[TABLE S2 HERE]

When we compare our sample to constituencies that are missing from the dataset in terms of socio-demographic and economic characteristics of the seats, we find that our sample tends to be more affluent and educated. Such a finding is consistent with the idea that individuals with more resources or interest in political matters are more likely to participate in politics. If such individuals are more inclined to upload their leaflets, then it is unsurprising that our dataset overrepresents constituencies with more affluent and educated populations.

We find, however, no meaningful differences between our sample of constituencies and those omitted from it in terms of political differences. We find no differences with regards to the margin of victory at the 2019 General Election or the proportion of seats won by the Labour Party, the Conservative Party, Liberal Democrats, etc. We also find no regional differences between our sample and the constituencies excluded due to the unavailability of data.

*Table S1.* Percentage of OpenElections leaflets that mention issue areas and tactical situation

|  | ElectionLeaflets.org  (single coding) | | OpenElections  (verified coding) | |
| --- | --- | --- | --- | --- |
|  | Per cent | N | Per cent | N |
| Economy | 66.7 | 752 | 71.4 | 75 |
| Education | 57.4 | 647 | 61.9 | 65 |
| Environment | 58.2 | 656 | 79.1 | 83 |
| Brexit/Europe | 68.5 | 772 | 74.3 | 78 |
| Health | 72.1 | 813 | 72.4 | 76 |
| Immigration | 6.6 | 74 | 7.6 | 8 |
| Welfare | 15.6 | 176 | 19.1 | 20 |
|  |  |  |  |  |
| Tactical Situation | 25.8 | 291 | 21.0 | 22 |

*Table S2*. Constituency sample representativeness

| Variables | Not included | Included | p-value |
| --- | --- | --- | --- |
| *Socio-demographic characteristics* |  |  |  |
| Population density (number per square km) | 19.0 | 22.1 | 0.14 |
| Professional occupations (% of population) | 30.0 | 31.9 | 0.00 |
| Routine/manual occupations (% of population) | 29.5 | 24.7 | 0.00 |
| Level 4 qualifications or above (% of population) | 25.8 | 28.4 | 0.00 |
| No qualifications (% of population) | 23.9 | 22.4 | 0.00 |
|  |  |  |  |
| *Region* |  |  |  |
| England | 0.85 | 0.84 | 0.75 |
| Scotland | 0.09 | 0.10 | 0.47 |
| Wales | 0.07 | 0.06 | 0.69 |
|  |  |  |  |
| *Election results (current)* |  |  |  |
| Margin of victory | 25.2 | 25.5 | 0.80 |
| Conservative Winner | 0.58 | 0.58 | 0.92 |
| Labour Winner | 0.32 | 0.32 | 0.96 |
| Lib Dem Winner | 0.02 | 0.01 | 0.46 |
| Other Party Winner | 0.08 | 0.09 | 0.58 |
| N | 392 | 240 |  |
